# Heredity

**Published:** 1896-02

**Authors:** 


### Heredity.

An interesting lecture on "heredity" was delivered the other day in Madras by Surgeon Major J. Smith. He defined "heredity" as a law amongst animals and plants which ensures that the young ones shall possess the peculiar qualities and properties of their parents. Heredity in the living world is what gravitation is in the non-living—the great steadier of things. It is the hand-maid of natural selection; and while natural selection affords an opportunity for the development of qualities that best fit living creatures for their environment, heredity ensures that these qualities shall be duly transmitted from parents to offspring. The lecturer regretted the general neglect of the law of heredity in the improvement of the European races, and concluded by giving his idea that the people of every nation should be classified carefully in regard to their fitness for marriage and to the particular ties into which the individuals should enter. This classification, the lecturer observed, should begin at an age below that at which choices usually are made, and approvingly spoke of the customs of Sparta of old in this matter.—*Indian Lancet.*

